# Model-based and model-free pain avoidance learning

**DOI:** 10.1177/2398212818772964

**Published:** 2018-05-10

**Authors:** Oliver Wang, Sang Wan Lee, John O’Doherty, Ben Seymour, Wako Yoshida

**Affiliations:** 1 Department of Neural Computation for Decision-making, Advanced Telecommunications Research Institute International, Kyoto, Japan; 2 Department of Biology, Stanford University, Stanford, CA, USA; 3 Department of Bio and Brain Engineering, Korea Advanced Institute of Science and Technology, Daejeon, Republic of Korea; 4 Division of the Humanities and Social Sciences, California Institute of Technology, Pasadena, CA, USA; 5 Computational and Biological Learning Laboratory, Department of Engineering, University of Cambridge, Cambridge, UK; 6 Center for Information and Neural Networks, National Institute for Information and Communications Technology, Osaka, Japan

**Keywords:** Decision-making, pain avoidance, reinforcement learning, uncertainty

## Abstract

**Background:** While there is good evidence that reward learning is underpinned by two distinct decision control systems – a cognitive ‘model-based’ and a habitbased ‘model-free’ system, a comparable distinction for punishment avoidance has been much less clear. **Methods:** We implemented a pain avoidance task that placed differential emphasis on putative model-based and model-free processing, mirroring a paradigm and modelling approach recently developed for reward-based decision-making. Subjects performed a two-step decision-making task with probabilistic pain outcomes of different quantities. The delivery of outcomes was sometimes contingent on a rule signalled at the beginning of each trial, emulating a form of outcome devaluation. **Results:** The behavioural data showed that subjects tended to use a mixed strategy – favouring the simpler model-free learning strategy when outcomes did not depend on the rule, and favouring a model-based when they did. Furthermore, the data were well described by a dynamic transition model between the two controllers. When compared with data from a reward-based task (albeit tested in the context of the scanner), we observed that avoidance involved a significantly greater tendency for subjects to switch between model-free and model-based systems in the face of changes in uncertainty. **Conclusion:** Our study suggests a dual-system model of pain avoidance, similar to but possibly more dynamically flexible than reward-based decision-making.

## Introduction

In contrast to reward-based decision-making, the underlying structure of aversive decision-making is less well understood. This is especially true in the case of avoidance learning, in which decisions are made that lead to the reduction, delay or omission of an otherwise expected punishment. The control of acquisition and expression of avoidance has been the topic of debate for decades, not least because of the difficulty of any single model to account for the range of experimental findings (Dinsmoor, 2001; Gillan et al., 2016).

In instrumental (‘operant’) reward learning, there is widely thought to be two distinct systems that interact to guide behaviour: a cognitive ‘model-based’ system which incorporates explicit knowledge of the structure of the reward environment (the contingencies between states, actions and their outcomes) and a computationally simpler ‘model-free’ system which simply learns the value of actions given different states, emitting habit-like responses (Dayan and Balleine, 2002; Dickinson, 1985; Dolan and Dayan, 2013). How these two systems interact and cooperate has been the focus of recent theoretical and experimental studies, and evidence suggests the existence of a competitive process by which the cognitive ‘model-based’ system exerts control when the outcomes of decisions are more uncertain, but that the ‘model-free’ habit system assumes control when outcomes become more predictable and it is safe to rely on a computationally less expensive system to guide behaviour (Daw et al., 2005). In a recent study, Lee and colleagues described behavioural and neural evidence that the balance of control between the two controllers was mediated by a flexible ‘arbitration’ mechanism based on the relative reliabilities of each system, estimated from prediction errors (Lee et al., 2014).

In the case of avoidance, evidence of two systems underlying control is somewhat less clear-cut. For instance, instruction alone is sufficient to acquire avoidance behaviour, and excessive avoidance training can lead to a resistance to extinction of avoidance when aversive events are no longer possible (Gillan et al., 2014). However, there have been few clear demonstrations that two distinct systems operate in parallel during avoidance, and it is not known whether they are subject to the higher control of an arbitrator similar to that of rewards. If it is, this would help resolve previous debate about the sufficiency of any one system to control avoidance in multiple situations, and provide important insight into disorders such as obsessive compulsive disorder (OCD), which are thought to result from excessive habitisation of avoidance behaviour (Robbins et al., 2012).

**Figure 1.**
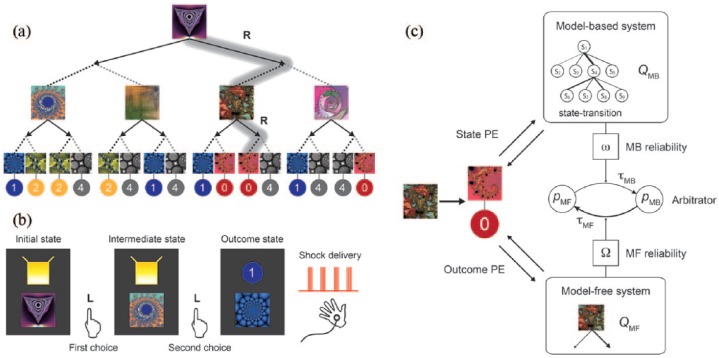
Two-step decision-making task. (a) Tree-shaped state mapping structure and an example state transition of a trial resulting from first choice (R: right) and second choice (R), and resulting in obtaining the 0 red coin. If the trial was in the flexible-goal condition, the subject would have received 0 shocks. If the trial was a specific-goal condition with a red collection box, the subject would also receive 0 shocks, but if the box was one of the other colours (blue or yellow), the subject would have received a default of 4 shocks. (b) Time sequence of a trial in the specific-goal condition. The initial, intermediate and outcome states are represented as a fractal image. With the decision states, the current goal is shown as a coloured box (e.g. yellow). After two consecutive choices, an outcome state is displayed with electrical shocks delivered on the back of the subject’s non-dominant hand. (c) Diagram of the computational model with arbitration between model-based and model-free learning systems.

We implemented a decision-making task to probe the balance of control between two putative systems of avoidance learning, mirroring a task paradigm and modelling approach recently developed for a financial reward task (Lee et al., 2014). Subjects performed a two-step instrumental avoidance paradigm in which an initial two-option decision could lead to one of four intermediate states (represented by visual images), and with a second two-option choice leading to an outcome state represented by a coloured visual image with an associated number of painful electrical stimuli (Figure 1(a)).

Before the experimental task, subjects performed a training session to learn about the states and their transitions, and hence the corresponding pain outcomes associated with different decision sequences. In the experimental task, subjects were required to try to avoid pain in two ‘goal conditions’. In a specific-goal condition, the outcomes were contingent on a rule: if the colour of the image that represented the outcome state matched that specified at the beginning of the trial, then the outcome state was delivered; if it did not match, the maximum pain was delivered. In a flexible-goal condition, the outcome state was delivered regardless of any rule relating to the colour of its representative image. The specific-goal condition requires cognitive representations (model) of pain outcomes and state transition probabilities (i.e. integrating the rule with explicit knowledge of the outcomes), whereas the flexible-goal condition can be solved by simple reinforcement of state-action sequences (habits). Following Lee et al. (2014), we proposed an arbitration model of avoidance learning in which two systems operate, with their contribution to a choice balanced by a reliability-based arbitration mechanism.

## Methods

### Subjects

A total of 15 subjects (2 females) participated in the study. All subjects were screened for a history of psychiatric or neurological problems, and were free of pain or pain medication. The subjects gave written informed consent before the experiment and the study was approved by the ethics committee of Advanced Telecommunications Research Institute International.

### Pain stimuli and calibration

The pain stimulus was delivered as a sequence of five square pulses of electric current (on for 2 ms and off for 8 ms) delivered by isolated bipolar constant current stimulator (DS7, Digitimer Ltd.) to a non-magnetic 5-mm diameter bipolar concentric electrode (Unique medical Co. Ltd) placed on the subject’s non-dominant hand (fascia over adductor pollicis muscle).

Before the experiment, subjects underwent a thresholding procedure to control for individual difference in skin resistance and pain tolerance. We administered the electric stimuli with gradual increases from 4 mA, and subjects provided visual analogue ratings of each shock on a scale from 0 (not painful) to 10 (intolerable pain). When subjects reported the strongest pain that they could tolerate, no higher stimulation was delivered. The level of shock delivered in the experiment was set to a stimulus strength of scale 5 (moderate pain) for each individual. Then the subjects were asked to rate 1, 2 and 4 sequential shocks, which are used as outcomes in the experimental task, to ensure they were ready and comfortable with the strength of stimulus. The average shock strength was 32.07 mA (standard deviation (SD) = 9.60).

### Experimental task

Subjects performed a two-choice decision-making task, in which they were required to make two sequential choices (by pressing ‘LEFT’ or ‘RIGHT’ button) to avoid a number of shocks at the outcome stage (after the second choice; Figure 1(a)). All possible states, one initial choice state, four intermediate choice states and four outcome states, were represented as different fractal images (Figure 1(a)). The outcome states were displayed on a coloured coin (red, yellow, blue and grey) with a numerical amount (0, 1, 2 or 4) that indicated the number of painful shock stimuli that subjects received as the outcome. In each trial, subjects began at the same initial state. Making no choice at either choice state within 4 s resulted in the computer making a random choice to proceed, and that trial was marked as a penalising trial and removed from the analysis. The inter-states and inter-trial intervals were sampled from a uniform distribution between 1 and 4 s. The shocks given at the end of a trial lasted 0.5 s and were administered with 0.4 s between subsequent shocks, followed by a 2-s delay with the state still on display.

Our experimental design involved performing the task in two different condition blocks: a ‘flexible-goal’ condition and a ‘specific-goal’ condition, with the conditions randomised between blocks. In both the conditions, a ‘collection box’ was displayed at each choice stage. In the specific-goal condition, the collection box had a specific colour (either red, yellow or blue, but not grey) and indicated the ‘goal’ on that trial (Figure 1(b)). In this condition, *only* the coin with the same colour as the collection box maintained its value, and the other coins were set the default maximum value of 4 shocks. That is, if the specific coloured coin was acquired, subjects receive the number of shocks associated with that coin, otherwise, they got 4 shocks regardless of the value stated on the acquired coin. During the specific-goal condition block, the goal (colour of collection box) was changed randomly from trial-to-trial. Thus, to avoid the maximum shocks, subjects had to continually consider which goal had avoidance value and plan their choices to acquire that goal, based on their learned knowledge of the action-outcome transitions. Note that the colour of the 4-shock coin was grey and this colour was never presented as the collection box (since this would result in 4 shocks regardless of choice). The specific-goal condition was thus designed to favour the model-based over the model-free control.

In the flexible-goal condition, a white collection box was presented, and subjects knew that all outcome coins retained their values, that is, in effect, the colour of the coins indicating the outcome value was irrelevant. In this condition, the best outcome is always the coins with no shocks (which happened to be the red coins) and there is no need to change their strategy on a trial-by-trial basis, and so in the situation, subjects can simply choose the best state-action contingencies and thus the model-free system should be dominant.

To further aid dissociation of model-based from the model-free control and to prevent subjects from using multiple model-free strategies in the absence of the model-based control in the specific-goal condition, two levels of uncertainty condition were used. In a low uncertainty condition the state transition probabilities to two consecutive states were 0.9 and 0.1, and in a high uncertainty condition the state transition probabilities were 0.5 and 0.5. Thus overall, there were four task conditions comprising two factors: specific- or flexible-goal and low or high uncertainty condition, and the order of four condition blocks was randomised. The blocks with low uncertainty consisted of three to five trials, whereas the high uncertainty blocks consisted of five to seven trials due to their difficulty. During each trial, subjects could recognise the goal condition but they were not explicitly told the state transition probabilities, so they did not know at the beginning of each block whether they were in the high or low uncertainty condition. The changes in the transition probabilities are rapid and are designed to induce perturbations in the predictions about state-transition probabilities, which in turn affected changes in the allocation of model-based and model-free control.

At the beginning of experiment, subjects performed a training session to learn about the states and corresponding pain outcomes. Importantly, however, subjects were not informed of the specific state transition probabilities used in the task; instead, they were told that the contingencies might change during the course of the experiment and that outcomes were not random. The state transition probability was fixed at 0.5 (high uncertainty) for all choice states and a white collection box was presented, indicating that any coin colour would lead to the indicated number of shocks (flexible-goal) though no shock stimulus was provided during training. The subjects performed 100 trials in the training session followed by two experimental sessions, each of which consists of 12 blocks of four task conditions, 48 blocks in total.

### Computational model

We applied the computational model proposed by Lee et al. (2014) to account for arbitration of two learning systems (Figure 1(c)). Accordingly, model-free and model-based learning systems jointly determine the final state-action value based on the reliabilities calculated from their recent prediction errors.

For the model-free learning system, a model-free SARSA, a variant of a classical reinforcement learning model (Sutton and Barto, 1998) was used. The model-free state-action value $Q_{MF}(s,a)$ defined in terms of the expected future outcome (reward or pain) from the current state s and action a. The value is updated by outcome prediction error (OPE), a temporal difference of actual outcome (*r*) and predicted immediate outcome at current state (difference in values of current state and action, $Q_{MF}(s,a)$, and next state and action, $Q_{MF}(s\prime ,a\prime )$), to decrease the inconsistencies

$$\begin{array}{l} \mathrm{OPE}=r+Q_{MF}(s\prime ,a\prime )-Q_{MF}(s,a)\\ \Delta Q_{MF}(s,a)=\alpha \mathrm{OPE} \end{array}$$

The model-based system learns both state transition probabilities and state-action value independently, but concurrently (Gläscher et al., 2010). This model constructs a FORWARD internal model of the state-transition matrix as T(s,a,s′) of state transition probabilities, which represents the probability of the agent arriving at state s′ if it made an action a in state s. When the agent made an action and observed the next state, the state transition is updated by the state prediction error (SPE), which is computed as a difference in the actual state transition and the estimated probability. With a given outcome r, the state-action value $Q_{MB}(s,a)$ is updated based on a dynamic programming method, that is, calculated as the product of the state-transition probabilities and the value (the sum of a given outcome and expected value of next state $Q_{MB}(s\prime ,a\prime )$)

$$\begin{array}{l} \mathrm{SPE}=1-T(s,a,s\prime )\\ \Delta T(s,a,s^{\prime })=\alpha \mathrm{SPE}\\ Q_{MB}(s,a)=\underset{s\prime }{\sum }T(s,a,s^{\prime })\left\{r+\max_{a\prime }Q_{MB}(s\prime ,a\prime )\right\} \end{array}$$

The learning rate of both model-free and model-based state-action value, *α*, is a free parameter and estimated from subjects’ actual behaviour. Here, we use a single learning rate for the model-free and the model-based as with Lee et al’.s reward-based learning task, because the two models’ performance difference is stable for a given learning rate that guarantees convergence. In addition, the model-based learning system in equipped with BACKWARD planning when the explicit goal condition is changed. In the specific-goal condition, this backward planning allows the system to devalue the outcome states (coins) whose colours are not the same as the ‘goal’ and also update the state-action values based on that.

The reliabilities of the two models are evaluated from the prediction errors, OPE and SPE, using a simple hierarchical empirical Bayes approach so that the larger prediction error decreases the reliability of the learning system. For the model-free system, the estimator ΩΩ learns to predict the absolute OPE as

ΔΩ=η−(|OPE|−Ω)

where η denotes a learning rate parameter for updating the estimation. If the estimator predicts zero OPE, the reliability of model-free system reaches the maximum. For the model-based system, on the other hand, if the SPE was less than a threshold parameter ω, the system counts the error was zero and the reliability reaches the maximum. To govern how two systems compete, a dynamical two-state transition model in which the states are the probabilities for choosing each system’s strategy, $p_{\mathrm{MF}}$ and $p_{\mathrm{MB}}$ (where $p_{\mathrm{MF}}=1-p_{\mathrm{MB}}$), was used. The transition rates between two states, $\tau_{\mathrm{MF}}$ and $\tau_{\mathrm{MB}}$, were a logit function, and modulated by the reliabilities. As the habits tend to emerge with increased training (Gläscher et al., 2010), there was a fixed bias to favour the model-free control if the reliabilities are equal.

Finally, the arbitration model calculates weighted state-action value of two systems and selects actions stochastically according to a soft-max function with an inverse temperature parameter β as (Gläscher et al., 2010; Luce, 1959)

$$\begin{array}{l} Q=p_{MB}Q_{MB}+p_{MF}Q_{MF}\\ P(s,a)=\frac{\exp\left(\beta Q\right)}{\sum \exp\left(\beta Q\right)} \end{array}$$

### Statistical analysis

Our model’s free parameters were fitted to the behavioural data by minimising the negative log-likelihood (−∑logP(s,a)) given the observed choices and rewards, summed across all subjects and trials. We used the Nelder–Mead simplex algorithm for optimisation (the function ‘fminsearch’ in MATLAB). To minimise the risk of local optima, we ran this optimisation 100 times with random seed parameters in the Linux-based high-performance computing (HPC) cluster environment. The implementation of the computational model of arbitration and all the statistical analyses were done with MATLAB 2014b.

Statistical comparisons between behavioural performances and parameter estimates were achieved with paired (compare between conditions) or two-sample (compare between subject groups) *t*-tests, implemented using the MATLAB Statistics and Machine Learning Toolbox.

## Results

**Figure 2.**
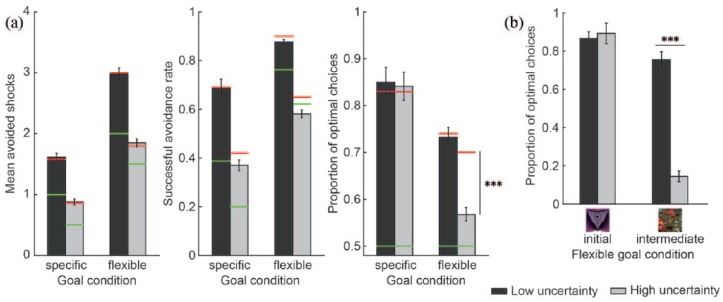
(a) Performance of the subjects in the form of the average number of shocks avoided, the successful rate of shock avoidance, and the proportion of optimal choices in the specific-goal (left bars) and the flexible-goal conditions (right bars). The dark and light grey colours correspond to the low and high uncertainty conditions, respectively. The red lines refer to the corresponding values of the reward-based task by Lee et al. The green lines define the baseline given by a random choice agent and illustrate the fact that avoiding shocks in the flexible-goal condition is easier than the specific-goal condition. Error bars are SEM across subjects. (b) In the flexible-goal condition, the proportion of optimal choices at initial state was not different between for both low and high uncertainty conditions, while they are different at the successive intermediate choice state. Error bars are SEM.

Figure 2(a) shows the avoidance performance of four task conditions, compared with the reward-based performance in Lee et al. (2014). The left panel shows the average number of shocks avoided on each trial, in relation to the comparable performance for reward in the previous study. Note that as the previous study used 10, 20, 30 and 40 cents for the monetary reward, here we show the reward values divided by 10 to compare the scale. The middle bar graph shows the success rate, defined as the proportion of trials avoiding pain, again compared with that of acquiring reward in previous reward-based task. The right panel illustrates the frequency of optimal choices, defined by the ideal agent’s behaviour in each condition. The baseline performance given by a random choice agent illustrates the fact that performance was inherently easier (i.e. could avoid more shocks) in the flexible-goal condition and in the low uncertainty condition. Overall, these results indicate that the subjects performed well across conditions, with clear sensitivity to goal condition and uncertainty. This suggests that pain avoidance learning shows a basic similarity in observed performance, with respect to sensitivity to condition and uncertainty, as reward learning.

However, one notable difference is apparent. In comparison with behaviour in the reward-based learning task, the optimal choice rate in the flexible-goal condition under high uncertainty was significantly lower in the pain-based learning task (Figure 2(a) right panel, *t*-test with unequal variance; *t* = 5.335, *p* = 6.16e^−06^), and successful avoidance rate was even marginally lower than baseline performance. In the flexible-goal condition, the optimal choice on the initial choice state was typically the same, that is, choosing Right, for both low and high uncertainty conditions, and the subjects performed equally well (Figure 2(b) right bars). But on the subsequent intermediate (second) choice state, the optimal choice in low and high uncertainty condition was different, that is, choosing Right and Left, respectively, and it was at this point where subjects make significantly more errors for pain compared to reward (Figure 2(b) left bars, paired *t*-test with equal variance; *t* = 9.777, *p* = 1.23e^−07^). Indeed, the frequency of optimal choices was significantly less than chance performance (*t* = 12.427, *p* = 5.97e^−09^).

**Figure 3.**
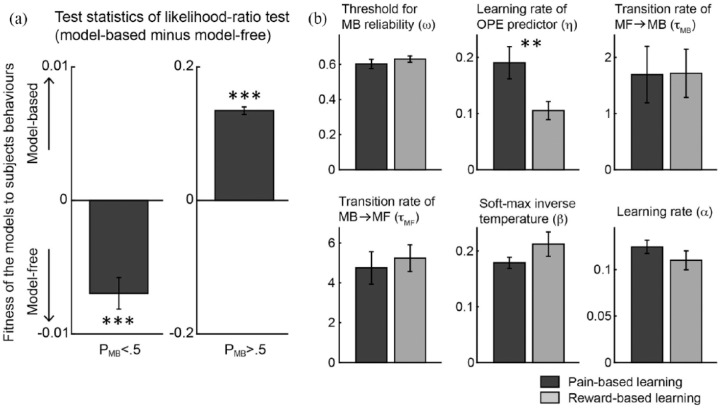
(a) Results from a log likelihood test comparing the degree to which model-based versus model-free reinforcement learning accounts best for subjects’ choices. Test statistics refer to log likelihood value of the model-based minus the model-free. When the arbitrator favours model-free control (left, *P*_MB_ *<* 0.5), the ratio was negative (*p* *<* 10^−3^), that is, the model-free system accounts better for behaviour. On the other hand, the ratio was positive for the situations in which model-based control was dominant (right, *P*_MB_ *>* 0.5) indicates that the model-based system was favoured (*p* *<* 10^−8^). Error bars are SEM. (Note: same analysis as Lee’s Figure 3(d).) (b) Comparison of model parameters between our pain-based learning task (left bars) and the identical reward-based task by Lee et al. (right bars). The figure shows six model parameters; the threshold for defining zero-state prediction error (for model-based reliability), the learning rate of the estimator of outcome (reward or pain) prediction error (for model-free reliability), the amplitude of transition rate function from model-based to model-free, the amplitude of transition rate from model-free to model-based, inverse softmax temperature and the learning rate. The learning rate of the outcome prediction error estimator was larger in pain-based task than that in reward-based task (*p* = 0.009).

To get more insight into the underlying decision-making architecture to account for the behaviour, we next applied a computational modelling approach to fit the choices to model-free and model-based decision-making algorithms. The more the arbitrator favours the model-based learning strategy, the less consistent subjects’ choices become. To provide a statistical measure of the model-based influence on choice, we used a likelihood-ratio test (Figure 3(a)) in which we separately fit the model-based and model-free algorithms to behaviour to prevent circularity and computed the log-likelihood value of the model-based minus model-free system. The more negative the ratio, the more the model-free system accounts better for behaviour, while the more positive the ratio the more the model-based system accounts better for behaviour. This analysis revealed that choice behaviour is better explained by the model-free learner in the flexible-goal condition when the arbitrator predicts that behaviour should be under model-free control (Figure 3(a) left bar, likelihood-ratio test; *p* *<* 10^−3^), while the choice behaviour is better explained by the model-based learner in the specific-goal condition when the arbitrator predicts that behaviour should be predominantly under model-based control (Figure 3(a) right bar, likelihood-ratio test; *p* *<* 10^−8^). These findings thereby validate the task manipulations by showing that the task can successfully manipulate control to be governed predominantly by either the model-based or model-free system.

Next, we compared the model parameters with those from Lee et al.’s otherwise identically structured task for rewards (Figure 3(b)). There are six parameters: (1) a threshold parameter for model-based reliability, (2) a learning rate for the estimate of absolute OPE for Bayesian update of model-free reliability, (3, 4) transition rates from model-free and model-based system and vice versa which control the competition on reliability-based dynamical transition system, (5) inverse soft-max temperature for action selection and (6) a learning rate for both learning systems. We found that the only parameter that differed between pain- and reward-based tasks was the learning rate for updating the prediction error estimator (Figure 3(b), *t*-test with unequal variance; *p* = 0.009), which controls how *quickly* the estimator updates the model-free reliability (note that the conventional value learning rate did not differ). The greater rate for punishment means that reliability is more dynamic (is more sensitive to short-term changes in outcome predictions) and so this will yield more dynamism in the transition between model-free and model-based systems.

## Discussion

The results yield three basic conclusions. First, they provide evidence that avoidance learning can be under the control of two different systems – a cognitive ‘model-based’ system and a ‘model-free’ habit system. Second, they suggest that the balance of each is under the control of a reliability-based arbitration mechanism. And third, they demonstrate a significant difference between reward- and punishment-based learning, in that there is greater dynamism in the transfer of control between model-based and model-free systems in avoidance.

The results provide insight into the nature of avoidance learning by formalising the concept of separable value systems governing behaviour. There has been a growing recognition that any comprehensive account of avoidance needs to accommodate both associative accounts (two-factor theory and safety-state reinforcement (Dinsmoor, 2001; Mowrer, 1947)) and cognitive accounts (for free-operant avoidance (Mineka, 1979)). The latter invoked more sophisticated internal representations of the structure of avoidance, incorporating the capacity to generate expectancies (Gillan et al., 2016; Seligman and Johnston, 1973). The results formally support a two-system account of avoidance, with the two systems reflecting the core difference between model-free and model-based learning: since the model-free lacks any way of incorporating the re-evaluation of outcome states given the outcome rule, it remains purely by the learned values of the cues at the decision points, that is, it cannot ‘see ahead’. Although there are slightly different computational ways of implementing a model-free system, all share this fundamental restriction. Similarly, the model-based learning systems should in principle encompass a broad array of environmental models, causal models and models over multiple timescales. Critically, they have the ability to re-evaluate their decisions based on rule-based information, and this allows them to behave optimally when prospective outcome states are revalued.

The results also suggest a flexible arbitration system in which the relative contribution of model-free and model-based controllers reflects an adaptive transition over a number of trials. The close similarity of the parameter estimates of the data in this study and our previous reward learning study raises an additional point of debate on the nature of avoidance learning – whether the underlying control architecture is the same or different. Whereas the nature of the reinforced outcome may be fundamentally different in avoidance (an excitatory reward state in appetitive reinforcement, and an inhibitory safety state in avoidance), this does not necessarily mean the control system that learns and emits the appropriate actions is different. Plenty of neural data show a common neuroanatomy of reward and avoidance actions (Kim et al., 2006; Park et al., 2011; Seymour et al., 2012; Talmi et al., 2009), but such data are purely correlative. Evidence from rodents suggests differences in the neural mechanisms of reward- and safety state-driven action reinforcement (Fernando et al., 2013); however, it is possible that this difference concerns the representation of the outcome state – pain relief versus reward, and not necessarily the control architecture that exploits it. A further uncertainty in this debate relates to whether there is any specific excitatory representation of an aversive action-outcome value for the avoided action (i.e. whether or not only reward-orientated action learning system is required, with aversive influences being purely Pavlovian, given the sophisticated array of Pavlovian aversive responses that emulate instrumental avoidance in most naturalistic situations (Bolles, 1970; Bolles and Fanselow, 1980).

While acknowledging the limitations of a single study with a modest sample size and biased gender distribution, the fact that all-but-one of the parameters is so similar across both reward and avoidance would seem to better support the existence of a common control architecture, but it also places the spotlight on the difference in the uncertainty update parameter. Here, we see that a difference exists in which system is judged to be more suitable. More specifically, the rate at which the model-free reliability is updated is greater in pain as opposed to reward. This renders the evaluation of model-free reliability more sensitive to short-term changes, meaning, for instance, that an unexpected sequence of large prediction errors will cause a more rapid transition to a model-based controller. With this in mind, it is likely that in some situations such enhanced dynamism may be sub-optimal. In particular, in the face of high uncertainty in the flexible control condition, the best strategy is in fact to stick with a model-free controller and ‘ride out’ the uncertainty, a fact that arises because the underlying state-transition probabilities are fixed (i.e. not-changing) in this task. However, switching more frequently to a model-based controller in the pain condition is likely to lead to behavioural changes that are not actually necessary; indeed it is likely to lead to switches early in uncertain blocks, leading to a greater proportion of suboptimal choices given the relatively short block afford little opportunity to find the correct behaviour. Therefore, it is likely that the sub-optimality we see in the data during avoidance relates to the different tuning of this reliability learning parameter. However, this somewhat counterintuitive behaviour should be further explored in future experiments and simulations. Also, an important caveat here is to note that the reward data comes from a scanner-based version of the task performed in a separate lab. Therefore, we cannot rule out the fact that contextual effects, such as that related to less anxiety in performing the task at a desk, do have the potential to confound any specific parameter. For this reason, the result should be cautiously interpreted and considered provisional in the absence of a within-experiment/subject contrast.

Finally, the data may have implications for our understanding of psychiatric disease, in particular OCD. It has been proposed that OCD might result from an excessive dominance of model-free avoidance – that is, avoidance habitisation (Robbins et al., 2012). In this framework, avoidance actions that may have at one time been ‘rational’ become ‘over-habitised’, because of subjects’ inability to transition back to a model-based controller that can explore the absence of punishment with different actions. Evidence for this comes from several experiments showing a resistance to extinction and devaluation in OCD patients in tasks inspired by a two-controller framework. OCD patients display reward-based equivalent behaviours (e.g. addictive behaviours) as well (Gillan et al., 2011), but there is currently no investigation that has directly compared excessive habit formation on avoidance and addictive behaviours in a systematic way. The typical compulsions in OCD are avoidant rather than appetitive, suggesting some independence of the mechanisms of reward and avoidance arbitration. Consequently, tasks such as the one presented here may offer a potentially useful future experimental approach for understanding distinct mechanisms of pathological behaviour: both to gain insight into OCD and as a general illustration of the value of the emerging field of computational psychiatry (Montague et al., 2012).
